# Potato cyst nematodes *Globodera rostochiensis* and *G*. *pallida*

**DOI:** 10.1111/mpp.13047

**Published:** 2021-03-11

**Authors:** James A. Price, Danny Coyne, Vivian C. Blok, John T. Jones

**Affiliations:** ^1^ School of Biology Biomedical Sciences Research Complex University of St Andrews St Andrews UK; ^2^ Cell & Molecular Sciences Department The James Hutton Institute Dundee UK; ^3^ International Institute of Tropical Agriculture (IITA) Nairobi Kenya

**Keywords:** genome sequence, *Globodera*, host–parasite interactions, potato cyst nematodes

## Abstract

**Taxonomy:**

Phylum Nematoda; class Chromadorea; order Rhabditida; suborder Tylenchina; infraorder Tylenchomorpha; superfamily Tylenchoidea; family Heteroderidae; subfamily Heteroderinae; Genus *Globodera*.

**Biology:**

Potato cyst nematodes (PCN) are biotrophic, sedentary endoparasitic nematodes. Invasive (second) stage juveniles (J2) hatch from eggs in response to the presence of host root exudates and subsequently locate and invade the host. The nematodes induce the formation of a large, multinucleate syncytium in host roots, formed by fusion of up to 300 root cell protoplasts. The nematodes rely on this single syncytium for the nutrients required to develop through a further three moults to the adult male or female stage. This extended period of biotrophy—between 4 and 6 weeks in total—is almost unparalleled in plant–pathogen interactions. Females remain at the root while adult males revert to the vermiform body plan of the J2 and leave the root to locate and fertilize the female nematodes. The female body forms a cyst that contains the next generation of eggs.

**Host range:**

The host range of PCN is limited to plants of the Solanaceae family. While the most economically important hosts are potato (*Solanum tuberosum*), tomato (*Solanum lycopersicum*), and aubergine (*Solanum melongena*), over 170 species of Solanaceae are thought to be potential hosts for PCN (Sullivan et al., 2007).

**Disease symptoms:**

Symptoms are similar to those associated with nutrient deficiency, such as stunted growth, yellowing of leaves and reduced yields. This absence of specific symptoms reduces awareness of the disease among growers.

**Disease control:**

Resistance genes (where available in suitable cultivars), application of nematicides, crop rotation. Great effort is put into reducing the spread of PCN through quarantine measures and use of certified seed stocks.

**Useful websites:**

Genomic information for PCN is accessible through WormBase ParaSite.

## Graphical abstract

PCN are biotrophic pathogens with a survival stage that allows them to persist in soil in the absence of a host facilitating their spread to all regions where potatoes are grown.

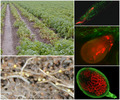

## 1 INTRODUCTION

Potatoes are among the most important staple food crops. They are a major source of carbohydrate and provide more calories, protein, and minerals than any other staple crop. An increase in the global consumption of potatoes, stimulated through rising populations and intensifying urbanization, has driven a surge in potato production (Birch et al., 2012). However, production is still adversely affected by pests and pathogens, including the potato cyst nematodes (PCN) *Globodera rostochiensis* and *G*. *pallida*. While these species can infect numerous species of *Solanum*, they are, in economic terms, principally parasites of potatoes (Whitehead, 1985).

Potato cyst nematodes originated in South America, where they coevolved with their solanaceous host plants. Although potato was first brought to Europe in the 16th and 17th centuries, it is likely that PCN were introduced from South America in the mid‐1800s on material for breeding resistance against *Phytophthora infestans* following the Irish potato famine (Evans et al., 1975). Genetic studies of PCN populations from across the world indicate that a relatively limited number of introductions of PCN into Europe have occurred (Blok et al., 1997; Hockland et al., 2012), with considerably greater genetic diversity in South American populations than those in Europe (Grenier et al., 2001). Analysis of mitochondrial *cytB* sequences of populations from Europe and South America identified a region in southern Peru from where the three distinct introductions of *G*. *pallida* in Europe are likely to have originated (Plantard et al., 2008). Europe has subsequently acted as a secondary distribution centre for PCN, most likely due to distribution of contaminated seed potato material (Hockland et al., 2012). Consistent with this, genotyping of populations occurring outside Europe shows “typical” European population types rather than exotic types from the centre of origin (e.g., Pylypenko et al., 2008).

The damage that PCN causes, and the huge difficulty in eradicating PCN once it is established in the field, have led to the implementation of strict quarantine regulations across many parts of the world that aim to contain and prevent the further spread of PCN. For example, within the EU, Council Directive 2000/29/EC permits member states to adopt quarantine measures that prevent the spread of PCN, while EU Council Directive 2007/33/EC describes measures for the control and management of PCN. More recently, this legislation has been updated with the implementation regulation 2016/2031 on Plant Health. Similarly, in North America, CIFA (Canada) and USDA‐APHIS (USA) developed guidelines on the application of sanitary and phytosanitary measures in accordance with the International Plant Protection Convention and the World Trade Organization Agreement. Soil samples are routinely collected and, on detection of PCN, movement of soil and crops (present and historic) from infected and adjacent fields is monitored and regulated until viable PCN are no longer detected. Infected land may not be used for host crop production unless PCN management plans are in place. Meanwhile in Australia, PCN biosecurity zones were established following detection of PCN in 1986 in Western Australia and in 1991 in Victoria. In New South Wales, biosecurity zones have been established to protect seed potato areas. Monitoring of this zone restricts import of potato propagative material, packaging, soil, and machinery from outside the area (Biosecurity Order 2017). Similarly, *G*. *rostochiensis* was identified in New York state in the USA in the 1940s but an aggressive programme of survey, quarantine, and deployment of resistant cultivars containing the *H1* gene have prevented further spread of this nematode (Evans & Brodie, 1980). Strict local and national import measures have led to the localized eradication of PCN though surveillance programmes continue to monitor for PCN. However, despite such stringent measures, new outbreaks of PCN are regularly reported, including in regions that are heavily dependent on potato production. For example, *G*. *pallida* was reported from Idaho in the USA, which is among the most important potato‐growing regions in the USA (Hafez et al., 2007), necessitating major efforts to contain and eradicate this outbreak (e.g., Contina et al., 2020). However, of potentially even greater significance is the discovery of PCN in a number of sub‐Saharan African countries (Cortada et al., 2020; Mwangi et al., 2015; Niragire et al., 2019).

**FIGURE 1:**
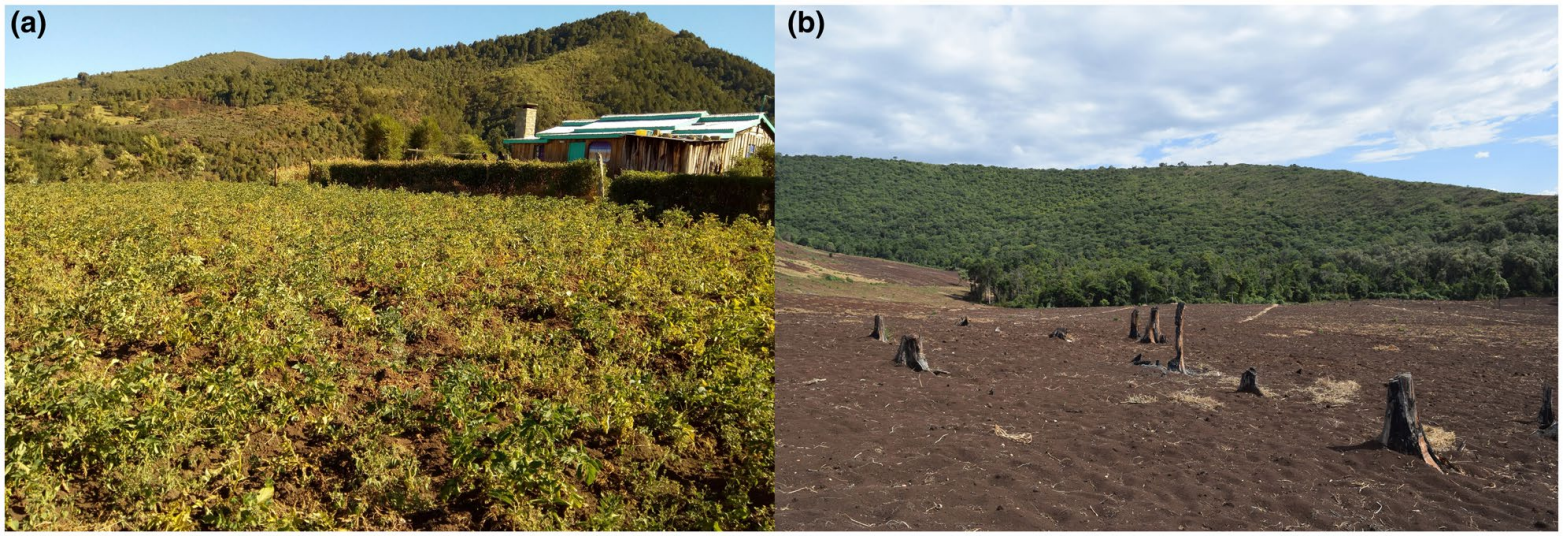
(a) Damage to potato crop in Kenya. Plants are stunted and show yellowing symptoms. Subsequent analysis showed that this field contained over 1,500 eggs per gram of soil. (b) Deforestation for potato production in sub‐Saharan Africa. The remnants of the first potato crop grown on cleared forest land are visible in the foreground

Potato is a crop of key importance in East Africa and the second most important crop after maize in many countries in the region (CIP, 2019). It is grown both as a cash crop and for food security. However, yields have precipitously declined in recent years to around 9–10 tonnes/ha (Kiptoo et al., 2016), considerably below the yield potential of this crop. Such yield losses are likely to be at least partly attributable to disease pressure. PCN (*G*. *rostochiensis*) was first reported from the region in Kenya (Mwangi et al., 2015) and subsequently shown to be widespread across the country, in over 80% of potato‐growing areas (Mburu et al., 2020), with *G*. *pallida* present in a small number of locations (Mburu et al., 2018, 2020). Subsequent surveys have identified *G*. *rostochiensis* in Rwanda (Niragire et al., 2019) and Uganda (Cortada et al., 2020), indicating a more region‐wide problem. Potato is cultivated in alignment with the two rainy seasons that occur each year, and mostly by smallholder farmers, who practise little or no rotation. These growing conditions, coupled with the fact that the most popular cultivars have no resistance against PCN, has proven ideal for PCN population densities to build up to extremely damaging levels; over 150 viable eggs per gram of soil have been recorded at multiple sites, while infestation levels that are considered low have around 30 viable eggs per gram of soil (Mburu et al., 2020). These infestation levels compare with fewer than 10 eggs per gram of soil in the UK, found in over 60% of infested fields (Minnis et al., 2002). Such high population levels are resulting in major yield losses and crop failures (Figure 1a; Mburu et al., 2020), with impacts at multiple levels. For example, persistent low yields push farmers to move to new land for potato production, opening up new fields even from forested areas. This food insecurity leads to deforestation therefore, as farmers shift from land deemed as unproductive (Figure 1b), with consequent environmental impact as forests are removed to make way for potato production.

## 2 CONTROL AND IMPACT OF PCN

The main strategies for the management of PCN can be separated into three categories: chemical, cultural (S.A.S.A., 2010), and natural host resistance. In addition, biological control methods, such as the use of antagonistic microbial agents, have been assessed and employed but are not widely used against PCN (reviewed by Davies et al., 2018). Few natural enemies of PCN have been identified and mass production of these is technically challenging (Kerry & Hominick, 2002; Viaene et al., 2006). No single strategy can be relied upon as none thus far is completely effective against both species. Many of the chemical nematicides previously used against PCN have also since been removed from the market due to their potential adverse environmental and human health concerns (Turner & Rowe, 2006). Just a few nematicides remain, such as Vydate (oxamyl) and Nemathorin (fosthiazate), although new chemistries are emerging onto the market that may prove effective with fewer nontarget consequences. The application of synthetic nematicide protects yield but results in significant additional costs to growers. In addition, timing of application needs to be carefully calculated in order for them to be effective. Cultural strategies attempt to limit the impact of PCN through improved agricultural practices. These may include extended rotations between potato crops to exploit the natural decline in PCN populations in the absence of a host, as well as the use of trap crops, biofumigation techniques, or the early destruction of susceptible potato crops before the PCN life cycle has completed. This latter technique, however, has a hugely detrimental impact on crop yield. Cultural control methods need to be very carefully monitored and as they are rarely 100% effective and can be costly to implement. Good farm hygiene, including cleaning of equipment and machinery, will help minimize spread between fields, while regular testing for the presence of PCN helps in monitoring for the pest, but for seed crop production it is essential to demonstrate that the land is PCN‐free. The most cost‐efficient and effective strategy is the use of natural host resistance (see below), which, in combination with other techniques such as chemical and cultural methods, is especially recommended as an integrated pest management system. However, effective deployment of resistance requires accurate species, and ideally pathotype, designation. Although the molecular factors that determine the virulence of PCN are not yet known, a high‐throughput tool for identification and genotyping of *G*. *pallida* populations has been developed based on MiSeq sequencing of pooled mitochondrial *cytB* sequences (Eves‐van den Akker, Lilley, Reid, et al., 2015). This has enabled the large‐scale genotyping of populations. Despite considerable efforts to control PCN, it continues to cause substantial yield losses.

Calculating the cost implications of PCN to growers is extremely challenging, given the uneven distribution of PCN and the varying impacts on yield for each different cultivar and soil type. Various figures have appeared in the literature, often with little detailed description of how they have been determined. A calculation based on a series of data (File S1) indicates that within the UK alone costs due to PCN may average £31 million per annum in relation to yield losses and management costs. This figure excludes losses incurred as a result of growing alternative, less profitable crops or due to the inability to use infested land for seed potato production and is therefore likely to be an underestimate of the economic impact of PCN.

## 3 LIFE CYCLE

Juvenile PCN develop within a chitinous eggshell. Following embryogenesis, the first moult occurs within the egg, giving rise to the second stage infective juvenile (J2), which then hatches from the eggshell. Hatching depends on many environmental factors, such as soil temperature and moisture, but most significantly it depends on chemical cues within host root exudates. Responding specifically to host exudates allows this host‐specific pathogen to coordinate its life cycle with the presence of a host. The detailed chemical composition of host root exudates is complex and remains to be properly characterized. The mixture of chemicals includes a range of secondary metabolites whose structure is often difficult to determine. PCN responds to numerous compounds present in root exudates (e.g., Byrne et al., 1998) as opposed to a single chemical component to induce hatch. To date, three key hatching stimulants have been identified from potato root exudates, solanoeclepin A (Tanino et al., 2011), α‐chaconine, and α‐solanine (Devine et al., 1996), while the steroidal alkaloids (aglycones) solanidine and solasodine, which are found in potato root exudates, will also stimulate hatch but to a lesser effect (Ochola et al., 2020).

Once hatched, infective J2 need to quickly locate host roots in order to feed before their reserves are depleted. They are guided to host roots by following chemical cues (Devine & Jones, 2003). After invading the root via intracellular penetration, the nematode migrates through host cells to the pericycle, where it identifies a cell suitable for transformation into a feeding site (the initial syncytial cell), usually in the inner cortex (Sobczak & Golinowski, 2011). The syncytium is formed by cell wall dissolution and fusion of protoplasts (Jones & Northcote, 1972). The J2 feeds on the syncytium contents using a feeding tube that is produced during each feeding cycle. It has been suggested that this acts as a filter to prevent destruction of the feeding site, which the nematode must keep alive for the duration of the life cycle (Eves‐van den Akker, Lilley, Jones, et al., 2015). Once the feeding site is established and feeding has commenced, the nematode develops through a further three moults to the adult stage.

**FIGURE 2:**
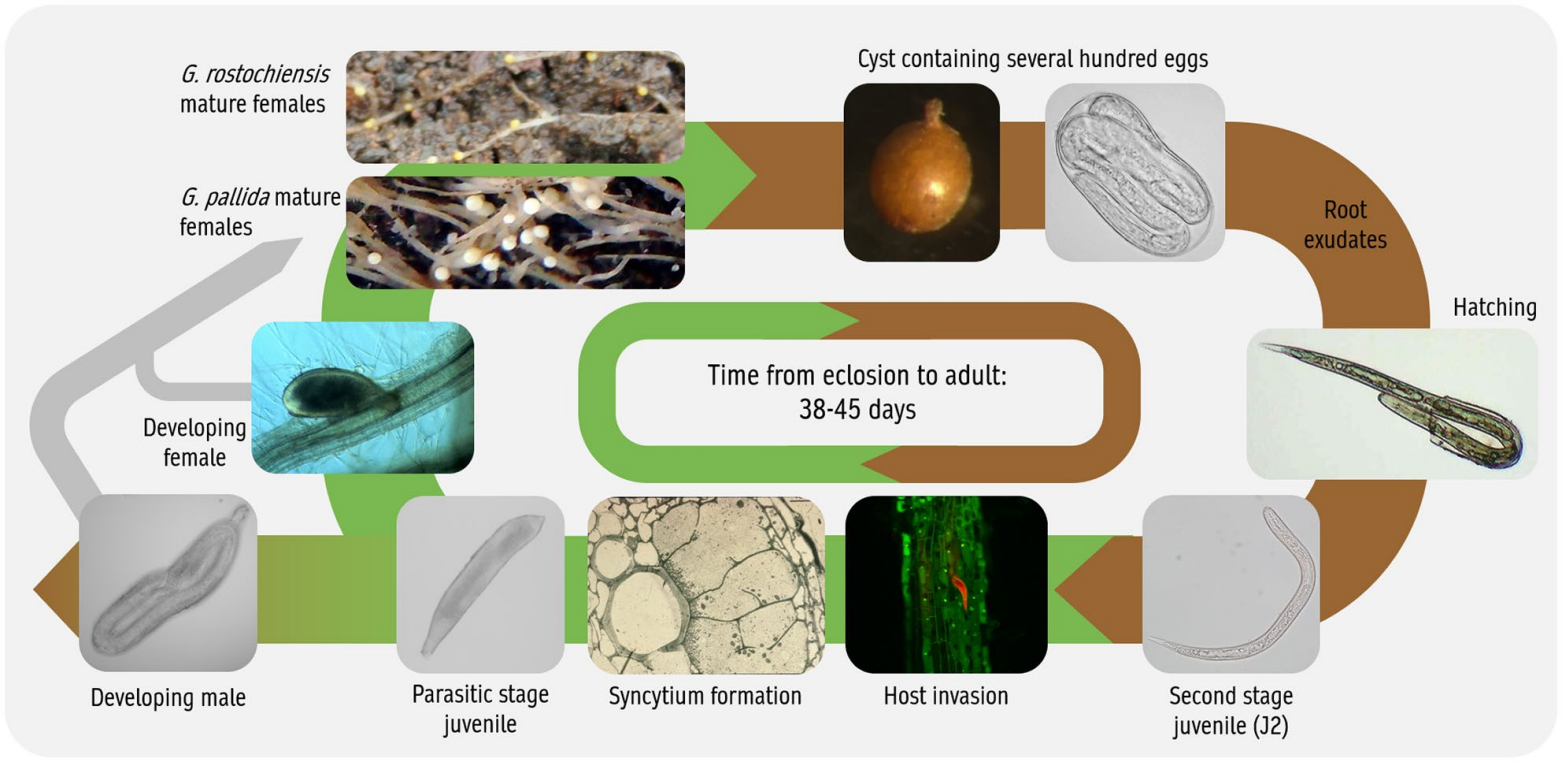
Key stages in the potato cyst nematode (PCN) lifecycle. Note that there is no difference between the overall life cycles of *Globodera pallida* and *G*. *rostochiensis*. Females of *G*. *pallida* are white, whereas those of *G*. *rostochiensis* are yellow

Although PCN reproduce sexually, the sex of the nematode is not genetically determined but instead is a response to external stimuli (Trudgill, 1967); in other cyst nematodes availability of nutrients has been shown to be a critical factor in sex determination (Grundler et al., 1991). Juveniles that successfully initiate a productive feeding site that abuts the vascular tissues of the plant tend to develop into females while those whose nutrient intake is restricted are more likely to develop into males. These nematodes may have induced feeding sites in suboptimal parts of the root or that fail to connect with the vascular tissues (Sobczak et al., 1997). Under high population densities, competition for nutrients can result in 10 times more males than females (Trudgill, 1967). Some natural resistance genes against PCN, including resistance derived from *Solanum sparsipilum* (Caromel et al., 2005), operate in this way, restricting development of the syncytium and thus resulting in a sex ratio skewed heavily towards males. Both males and females enter adulthood after the fourth moult. At this stage, the female has swollen so that her body ruptures the cortex of the root. Males exit the root and are attracted to the adult female due to pheromones that she releases (Green, 1980; Green & Plumb, 1970). As the swollen gravid female begins to senesce, her internal organs begin to deteriorate and the cuticle tans, hardens, and forms a toughened cyst wall. The cysts remain in the soil where J2 develop inside the eggs awaiting the next suitable host to stimulate hatch. PCN eggs can survive in a dormant stage inside the cyst for many years (Perry, 2002), meaning that eradication of the pest once soils are infested is extremely challenging. The life cycle of PCN is summarized in Figure 2.

## 4 GENOME AND TRANSCRIPTOME RESOURCES

The past decade has seen huge developments in our understanding of how plant‐parasitic nematodes (PPN), including PCN, interact with their hosts. Genome sequences for both *G*. *rostochiensis* and *G*. *pallida* are now available, and both these sequencing projects have included extensive transcriptomic analyses that have allowed identification of genes that play key roles in the interactions of PCN with their hosts (Cotton et al., 2014; Eves‐van den Akker et al., 2016). On the plant side of the interaction, much has been learned about the processes underlying development of the syncytial feeding structure induced by cyst nematodes, although much of this is underpinned by studies using a related cyst nematode, *Heterodera schachtii*, which can infect *Arabidopsis*.

Like many other PPN, PCN are challenging experimental organisms to work with as they are obligate biotrophs with relatively long life cycles and no large‐scale in vitro culture system is available. The life stages that interact with the host are embedded in the roots and are microscopically small. The challenges of working with PPN means that our understanding of the basis by which they interact with plants has generally lagged behind that of other important plant pathogen groups. The application of genomics and transcriptomics to these nematodes has provided a blueprint for a more detailed analysis of their biology.

Genome sequences for *G*. *pallida* (Cotton et al., 2014) and *G*. *rostochiensis* (Eves‐van den Akker et al., 2016) have now been assembled. Both were sequenced using (mainly) Illumina short‐read technology on UK populations. The sequence assembly for *G*. *rostochiensis* is of considerably better quality, in terms of fragmentation, number of unknown bases, and completeness as assessed using BUSCO/CEGMA analysis, than that of *G*. *pallida*. A greatly improved assembly for *G*. *pallida*, generated using a combination of PacBio and Illumina sequence reads, is, however, nearing completion (authors’ unpublished data). In general, the overall genome features for both species (genome size and proportion of repetitive elements) are broadly similar and it is likely that the differences in original assembly quality reflect the fact that the *G*. *pallida* populations are derived from a much larger and more genetically diverse original introduction than that which occurred for *G*. *rostochiensis* (Eves‐van den Akker et al., 2016).

The genomes of *G*. *pallida* and *G*. *rostochiensis* are approximately 120 and 100 Mb, respectively, similar to that of other cyst nematodes, such as *Heterodera glycines* (Masonbrink et al., 2019) and *Globodera ellingtonae* (Phillips et al., 2017). As yet, no evidence for the types of hybridization events and resulting polyploidy that have occurred in root‐knot nematodes (Blanc‐Mathieu et al., 2017; Eves‐van den Akker & Jones, 2018) has been found within the cyst nematodes. As for other plant and animal parasitic nematodes, fewer genes are predicted in PCN genomes compared with free‐living nematodes (Kikuchi et al., 2017).

One of the unusual genome features of PPN is the presence of genes acquired by horizontal gene transfer from bacteria and in one case from fungi (Kikuchi et al., 2004). For PCN this includes a wide range of cell wall‐degrading enzymes (Danchin et al., 2010) and cell wall‐modifying proteins (Qin et al., 2004) that are secreted during migration and which help soften the plant cell wall. Other horizontally acquired genes are also present in PCN, including a chorismate mutase, which may suppress host defences by preventing synthesis of salicylic acid (Jones et al., 2003), and a GH32 invertase, which is involved in the digestion of sucrose (Danchin et al., 2016). These horizontally acquired genes become homogenized once incorporated into the genomes of the nematodes; they contain multiple spliceosomal introns and are indistinguishable from other nematode genes in terms of GC content or codon usage (reviewed in Kikuchi et al., 2017).

**FIGURE 3:**
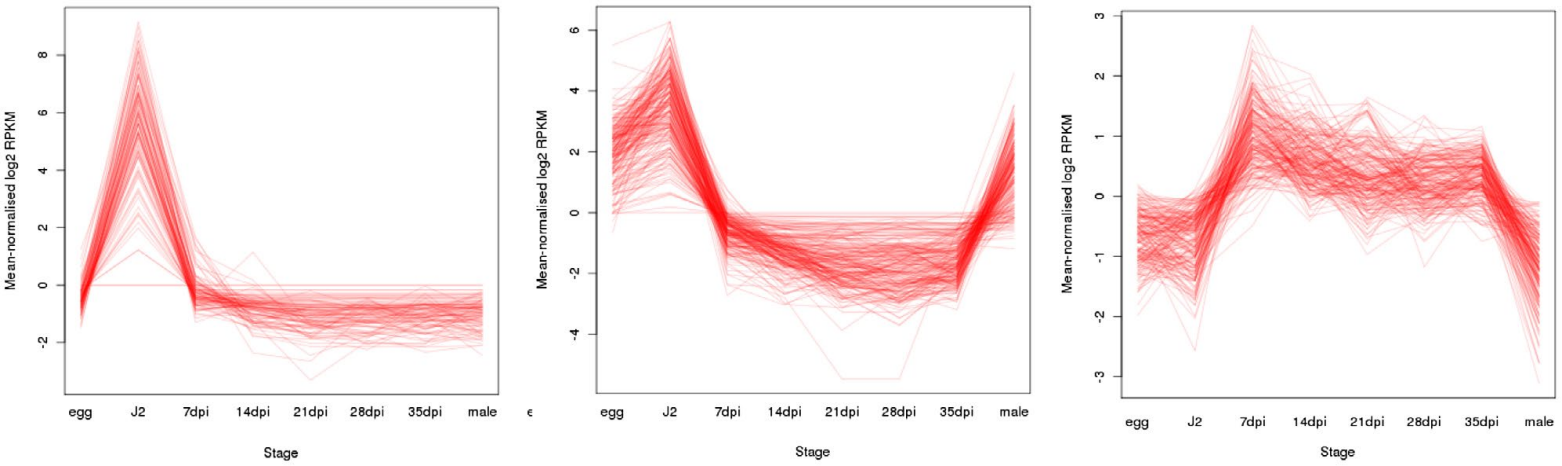
Clusters of genes from *Globodera pallida* showing common expression profiles at J2 (left hand panel), J2 and adult male stages (central panel), or parasitic stages (right hand panel)

The genome projects for both species of PCN have included comprehensive transcriptome analyses of gene expression across the life cycle. For *G*. *pallida* this encompassed eight different stages in the life cycle, while fewer life stages were analysed for *G*. *rostochiensis*, based on the findings from *G*. *pallida*. Analysis of the *G*. *pallida* transcriptome data showed that a limited number of distinct groups of life stages were distinguishable—eggs, J2, early parasitic, late parasitic and adult male—in which similar patterns of up‐ or down‐regulation of gene expression were observed. More detailed cluster analysis of the transcriptome data (Figure 3) showed that concerted changes in gene expression reflected the biology of these organisms; clusters of genes up‐regulated at parasitic (feeding) stages were enriched for genes encoding digestive enzymes. Genes encoding cuticle collagens were also enriched in such clusters, reflecting the onset of moulting in the feeding stages. Such analyses allow genes important in specific biological processes to be targeted for further analysis.

## 5 EFFECTORS OF PCN

One of the key outcomes of the genome/transcriptome projects for PCN has been the ability to generate comprehensive lists of candidate effector sequences using bioinformatic approaches that can subsequently be tested in the laboratory. Effectors of PPN, including PCN, are mostly expressed in either the subventral or dorsal pharyngeal gland cells (Jones et al., 2009). This restricted expression profile allows candidate effector sequences to be verified using in situ hybridization, which examines the spatial expression patterns of the candidate genes in the nematode. Initially, candidate effectors were identified on the basis of the presence of a signal peptide, the absence of a transmembrane helix, and significant up‐regulation in one or more parasitic stages of the nematode (Thorpe et al., 2014). Subsequently, an approach was developed for effector identification based on the presence of a promoter region previously identified as being associated with effectors known to be expressed in the dorsal gland cell of PCN (Eves‐van den Akker & Birch, 2016; Eves‐van den Akker et al., 2016). For this approach, the regions upstream of a collection of genes that had previously been experimentally verified as being expressed in the dorsal gland cell were examined using motif analysis software to identify motifs enriched in these genomic regions. One motif, the DOG box, was preferentially enriched in these regions as compared to a selection of known noneffectors and effectors expressed in the subventral gland cells. A statistical analysis of the entire gene set of *G*. *rostochiensis* showed that increased iterations of the promoter were associated with an increased probability of the motif being upstream of a predicted secreted protein. Functional validation of this promoter as a predictor for effectors was provided by the identification of new effectors based on the presence of the promoter (Eves‐van den Akker et al., 2016). Further analysis based on these approaches has shown that, as is the case for other biotrophic plant pathogens, several hundred potential effector sequences are present in PCN.

Morphological studies have shown that the subventral and dorsal gland cells of PPN are active at different stages of the life cycle (Hussey & Mims, 1990). The subventral gland cells show signs of being most active at the preparasitic J2 stage and are also active in adult males, while the dorsal gland cell grows throughout the life cycle. In keeping with this, analysis of the expression profiles of all predicted effectors of *G*. *pallida* showed that most of these sequences were expressed either at the J2 (= early parasitic; subventral gland cells) or parasitic (= later parasitic; dorsal gland cell) stages of PCN (Thorpe et al., 2014). Similarly, effectors used in the early stages of the life cycle, such as the cell wall‐degrading enzymes, which are used during invasion and migration are expressed primarily on the subventral gland cells (e.g., Smant et al., 1998). By contrast, effectors expressed in the dorsal gland cells have roles in the later stages of the interaction with the host plant, including suppression of host defences and induction of the feeding site (e.g., Lee et al., 2011; Postma et al., 2012).

One of the surprising findings emerging from the analysis of effectors in PCN is the presence of a high number of highly expanded effector gene families, most notably the glutathione synthetases and SPRY (SP1a and Ryanodine Receptor) domain proteins. SPRY domain proteins are ancient in evolutionary terms and are present in animals, plants, and fungi. The SPRY domain itself was originally identified in the spore lysis SP1a protein from the amoeba *Dictyostelium discoideum* (slime mould) and in mammalian Ca^2+^‐release channel Ryanodine receptors (Ponting et al., 1997). The SPRY domain is thought to be important in protein–protein interactions and no enzymatic activity has been identified that is associated with this domain (Perfetto et al., 2013). Most nematode species analysed to date have a relatively small number of SPRY domain proteins that do not have a signal peptide and are therefore predicted to be intracellular proteins. However, PCN possess greatly expanded SPRY domain protein families, including a subset (approximately 10%) that have a signal peptide for secretion and that are expressed in the dorsal gland cells (Cotton et al., 2014; Mei et al., 2015). Those SPRY domain proteins with a signal peptide (SPRYSECs) are expressed solely at the early stages of parasitism, while those without signal peptides either are not expressed or are expressed across the life cycle (Mei et al., 2015). Several studies have shown that at least some of the SPRYSEC proteins suppress effector‐triggered immunity (ETI) induced by the activation of resistance proteins (Ali, Magne, Chen, Obradovic et al., 2015; Mei et al., 2015; Postma et al., 2012). More recently it has been shown that one SPRYSEC targets the plant ubiquitination machinery to modulate stress responses (Diaz‐Granados et al., 2020). Although a role for SPRYSECs in suppressing host defence responses has been established, there are many questions remaining about this gene family. Why is this gene family so hugely expanded in cyst nematodes? What are the roles (if any) of the nonsecreted SPRY domain proteins, or do these sequences represent an “effector graveyard” of sequences that may be disadvantageous to the nematode? One SPRYSEC (RBP‐1) is recognized by a PCN resistance gene (*Gpa2*) (Sacco et al., 2009), indicating that these proteins play a role at the interface between plant and pathogen, and are therefore under strong selection pressure.

SPRYSEC effectors most probably evolved from a housekeeping protein present more widely in nematodes, as is also the case for the glutathione synthetase (GS) effectors. Glutathione, the tripeptide γ‐l‐glutamyl‐cysteinyl‐glycine, is an important antioxidant in plant and animal cells. It is synthesized in a two‐step process, with the final step being the addition of glycine to γ‐glutamylcysteine, catalysed by GS. Most animals analysed to date have a single GS present in their genomes, which encodes an intracellular housekeeping enzyme. However, in addition to this housekeeping sequence, cyst nematodes and related species have evolved a greatly expanded gene family of GS‐like sequences, whose proteins are secreted from the dorsal gland cell and are deployed as effectors during parasitism (Lilley et al., 2018). Structural and biochemical analyses have shown that the proteins have become diversified in their biochemical activity, presumably to play a key role in the parasitic process. In keeping with this, high levels of novel thiols were detected in the feeding structures of cyst nematodes (Lilley et al., 2018).

Other highly expanded families of candidate effectors are also present in PCN but for which no functional data are available. For example, a substantial gene family is present in *G*. *pallida* similar to the 4D06 sequences identified in *H*. *glycines* and *G*. *rostochiensis* (Ali, Magne, Chen, Côté et al., 2015; Gao et al., 2003), as well as a gene family of sequences similar to the “1,106” sequences from *G*. *rostochiensis* (Thorpe et al., 2014). Such highly expanded effector gene families seem to be a feature specific to cyst nematodes, as compared to root‐knot nematodes.

## 6 MOLECULAR BASIS OF THE COMPATIBLE INTERACTION WITH THE HOST

Cyst nematodes induce the formation of a remarkable syncytial feeding structure in the roots of their host plants. The morphological changes that underlie syncytium development in susceptible plants have been described in some detail (Sobczak & Golinowski, 2011). Following root invasion, J2 of PCN migrate intracellularly and destructively until they reach the inner cortex layer. At the inner cortex the behaviour of the nematode changes markedly; rather than using the stylet to destroy cell walls, the nematode uses this structure to probe individual cells, seeking a cell that does not respond adversely (either by collapsing or depositing callose). Cell wall openings in this initial syncytial cell are formed, initially by widening of pre‐existing plasmodesmata, followed by controlled breakdown of the plant cell wall in these regions. Numerous ultrastructural changes associated with the transformation of this cell into a much more metabolically active structure are then observed: the cytoplasm proliferates, the central vacuole breaks down, and the nucleus becomes enlarged. These changes are also observed in the cells surrounding the initial syncytial cell. Eventually the protoplasts of the initial syncytial cell and its neighbours fuse at the cell wall openings, a process repeated with further layers of cells until several hundred cells are incorporated into the syncytium. Changes in the expression of cell cycle genes occur in the syncytium, including DNA replication through endoreduplication. The process of syncytium induction is critical to the nematode; each nematode can only create one syncytium, which it is then dependent upon for all its nutrient requirements for development to the adult stage. The syncytium must therefore be maintained and kept alive for 4–6 weeks, a period of biotrophy that is almost unparalleled in plant–pathogen interactions.

Although we still do not have a detailed understanding of the molecular processes underlying syncytium induction by nematodes, significant progress has been made on two main fronts: the role for nematode CLE peptides and the importance of disruption of auxin transport. Cyst nematodes use CLAVATA3/ENDOSPERM SURROUNDING REGION‐related (CLE) peptide mimics to modulate host developmental programmes for feeding cell formation. Genes encoding CLE‐like peptides have been found in a wide range of cyst nematode species, including PCN (Lu et al., 2009), which are adapted to the specific host (Pokhare et al., 2020). Cyst nematode CLEs have a signal peptide for secretion from the dorsal gland cell, but also include a variable domain that includes a translocation signal that allows them to be localized to the plant endoplasmic reticulum for posttranslational modification and secretion from the plant cell into the apoplast (Wang et al., 2010, 2021). One of the *G*. *rostochiensis* CLE peptides binds with high affinity to the CLV2 receptor, which is itself up‐regulated at the nematode feeding site (Chen et al., 2015). CLE peptide receptors are receptor kinases that control a wide range of key plant developmental processes, including stem cell specification, organogenesis, and vascular differentiation (Guo et al., 2011). Manipulation of these endogenous pathways through CLE effectors may therefore play a key role in syncytium development.

Auxins are essential plant hormones that determine many plant developmental processes. It has been clear for many years that auxin signalling plays a critical role in the development of cyst nematode syncytia. Auxin‐insensitive mutants display inhibition or disruption of PCN syncytium development and disturbing polar auxin transport gives rise to abnormally large syncytia (Goverse et al., 2000). This study also showed that an auxin‐responsive promoter is specifically activated in developing syncytia. The mechanisms by which these changes in local auxin concentrations are achieved in syncytia have been analysed in some detail, in the context of the interactions between *H*. *schachtii* and *Arabidopsis*, and these studies indicate that auxin transport proteins play a critical role (Grunewald et al., 2009). Expression of *pin1* and *pin7* was shown to be down‐regulated in developing syncytia and development of nematodes on *pin1* mutant plants was severely impaired. By contrast, PIN3 accumulates in the syncytium but, remarkably, becomes relocalized within the syncytium, from a basal to a lateral site in the cell wall. These data indicate that auxin concentrations accumulate in the developing syncytium due to down‐regulation of *pin1*, preventing transport of auxin out of this structure. Subsequently, the relocalization of PIN3 allows auxin to be transferred to lateral cells surrounding the initial syncytial cell, permitting the radial expansion of this structure. Although it remains unclear as to how these changes in auxin transporters are achieved by cyst nematodes, one cyst nematode effector (19C07) has been identified that interacts with, and modulates, the activity of the LAX3 auxin influx transporter (Lee et al., 2011). LAX3 is usually expressed in cells surrounding lateral root primordia and allows influx of auxin, which subsequently activates the expression of cell wall‐modifying enzymes required for lateral root emergence. It is known that development of the syncytium requires controlled activation of the plant's own cell wall‐degrading enzymes to allow fusion of protoplasts of neighbouring cells (Goellner et al., 2001). LAX3 is expressed in developing syncytia, as well as in cells that are to be incorporated into syncytia, while the interaction between LAX3 and 19C07 takes place at the plasma membrane (Lee et al., 2011). Although *lax3* mutants are not significantly different to wild‐type plants in terms of susceptibility to nematode infection, *aux1*/*lax3* double mutants and *aux1*/*lax1*/*lax2*/*lax3* quadruple mutants show reduced infection levels, indicating the importance of the auxin influx process to syncytium development. In addition, overexpression of 19C07 speeds up lateral root emergence, indicating a probable stimulation of LAX3 by the effector. Although the function of the 19C07 has only been studied in detail in the *Arabidopsis–H*. *schachtii* pathosystem, similar sequences are present in PCN (Thorpe et al., 2014).

## 7 RESISTANCE AGAINST PCN

The use of natural host resistance has so far provided the most effective tool for management of PCN, particularly *G*. *rostochiensis*. The restricted genetic heterogeneity of the *G*. *rostochiensis* present in Europe means that durable resistance has been provided against this species in Europe with cultivars containing the *H1* gene. The *G*. *pallida* present in Europe is more genetically diverse and thus identification of a single major resistance (*R*) gene for control of this species has been more challenging (Caromel et al., 2005; van der Voort et al., 1997; van der Vossen et al., 2000). Progress has been made with processing cultivars such as Innovator, which contains the *Gpa5* gene from *Solanum vernei*. However, Innovator is intolerant and thus does not perform well where higher population densities of PCN are present and breakdown of this resistance has already been observed (Mwangi et al., 2019). Much effort is being given to address the lack of *G*. *pallida*‐resistant cultivars with appropriate agronomic characters. It is likely that these will require more considered management to preserve their durability. Resistance sources from *S*. *vernei* (*Gpa5*) and *S*. *tuberosum* subsp. *andigena* (*H3*) are widely used in commercial breeding programmes for *G*. *pallida*. The *S*. *vernei Gpa5* source is controlled by two quantitative trait loci (QTLs) that are located on chromosomes V and IX (Bryan et al., 2002), while the *H3* source is controlled by QTLs on chromosomes IV and XI (Bryan et al., 2004). Although these are the most commercially important of the *R* genes used for the control of *G*. *pallida*, the major QTL for neither has been cloned to date, although they have been mapped and molecular markers to assist in breeding selection are available (Moloney et al., 2010). Three PCN *R* genes have been cloned, *Gpa2* (van der Vossen et al., 2000), *Gro1‐4* (Paal et al., 2004), and *Hero* (Ernst et al., 2002). *Gpa2* is overcome by almost all European populations of *G*. *pallida* other than a single population (D383) from the Netherlands. *Gro1‐4* provides resistance against the Ro1 pathotype of *G*. *rostochiensis* only. By contrast, *Hero* provides broad‐spectrum resistance against all *G*. *rostochiensis* pathotypes tested and partial resistance against a wide range of *G*. *pallida* populations (Sobczak et al., 2005).

The resistant response of plants against PCN exploits the dependency of the nematode on the syncytium to provide the food required for its development. Each nematode can only induce a single syncytium and destruction of the syncytium, therefore, inevitably leads to death of the nematode. However, for PCN, it is rare for the hypersensitive reaction to be targeted at the syncytium itself but more common for the response to be seen in the cells surrounding the syncytium. For example, although the resistant response generated by *H3* may lead to syncytial degradation, the earliest phases of the response prevent expansion of this structure towards the vascular tissues (Varypatakis et al., 2020). Similarly, syncytia are induced in potatoes containing *H1* after infection by avirulent *G*. *rostochiensis* but development of these syncytia is restricted due to necrosis in cells surrounding the developing syncytia (Rice et al., 1985).

Almost all of the PCN resistance genes cloned to date are of the typical nucleotide‐binding leucine‐rich repeat (NB‐LRR) type of resistance gene. *Gro1‐4* encodes a NB‐LRR protein with a Toll‐interleukin receptor (TIR) domain at the N‐terminus, while *Hero* and *Gpa2* encode CC‐NB‐LRR proteins, which have a coiled‐coil (CC) domain at the N‐terminus. However, more recently, the *Cf‐2* gene, originally identified on the basis of its ability to confer resistance against the fungal pathogen *Cladosporium fulvum*, has been found to provide resistance against *G*. *rostochiensis*. *Cf‐2* encodes an extracellular receptor‐like protein with an LRR domain (Lozano‐Torres et al., 2012) and senses pathogen attempts to interfere with the apoplastic cysteine proteinase Rcr3, mediated in *C*. *fulvum* by the *Avr2* effector (Rooney et al., 2005) and in *G*. *rostochiensis* by the VAP‐1 effector. The targeting of the Rcr3 proteinase by both fungal and nematode pathogens shows that pathogens independently target the same potential host proteins and that by guarding such targets, plants can expand the range of pathogens recognized by their immune receptors.

Studies on resistance against the soybean cyst nematode, *H*. *glycines*, have shown that some resistance against cyst nematodes may be underpinned by entirely novel molecular mechanisms. In these cases, the underlying mechanism of resistance is based on a lethal toxic effect exerted through the feeding structure. Resistance provided through the *rhg1* locus is based on an αSNAP protein that accumulates preferentially in the nematode feeding site and has amino acid differences that cause a failure of its normal function (Bayless et al., 2016). Similarly, *rhg4* resistance is mediated through a variant form of serine hydroxymethyltransferase (SHMT) that impedes the development of the feeding site, causing a failure of nutrient supply to the nematode (Liu et al., 2012). These unusual resistance mechanisms show that plants have been able to evolve novel ways of combating PPN based on the reliance that these pathogens have on the feeding structure. Although no similar mechanisms against PCN have been identified to date, it is important that studies aimed at identifying resistance sources against PCN do not focus solely on NB‐LRR genes when genetic loci are mapped.

Recognition of PCN by host defences, including resistance genes, seems to be based on similar principles as for other biotrophic plant pathogens. Two candidate pathogen‐associated molecular patterns (PAMPs) have been identified from PPN (Manosalva et al., 2015; Mendy et al., 2017), although neither has been characterized in PCN. In addition, a potential pattern recognition receptor (NEMATODE‐INDUCED LRR‐RLK 1; NILR1, At1g7436) associated with detection of *H*. *schachtii* in *Arabidopsis* has been identified (Mendy et al., 2017). One PCN avirulence (Avr) gene, *AvrGpa2*, has been identified to date (Sacco et al., 2009). *AvrGpa2* is a SPRYSEC effector protein (RBP1) and recognition/loss of recognition is determined by a single amino acid polymorphism. Further analysis of sequence variation within RBP1 shows that this site is under strong selection pressure and is the only variable site that determines pathogen recognition (Carpentier et al., 2012). Further analysis of PCN using a variety of approaches has been undertaken to identify the genetic differences underlying recognition by the host, or that underpin the ability to infect particular host species. For example, several attempts have been made to identify transcriptomic differences in PCN that are associated with differences in host range. Comparisons of genes expressed in PCN with two closely related species that have a different host range, *G*. *tabacum* and *G*. *mexicana*, showed a strong enrichment for effector‐encoding genes amongst the differentially expressed sequences (Sabeh et al., 2019). Genomic approaches have also identified differences in effector sequences as potentially being critical in underpinning differences in virulence, in this case defined as the ability to reproduce on plants carrying a particular resistance source. Several studies of genomic differences between lines of *G*. *pallida* selected for virulence against the *H3* resistance source from *S*. *tuberosum* subsp. *andigena* or the *S*. *vernei Gpa5* source showed that sequences encoding potential effectors, particularly SPRYSEC effectors, were among the most likely to show polymorphisms (Eoch‐Bosy et al., 2017; Varypatakis et al., 2020). The latter study took advantage of a greatly improved genome assembly for *G*. *pallida* to identify specific genome regions that preferentially accumulated polymorphisms in response to selection pressure (Varypatakis et al., 2020).

## 8 DEPLOYMENT OF RESISTANCE AGAINST PCN

Natural resistance remains the most sustainable and cost‐effective method for the management of PCN. Compared to aerial plant pathogens, PCN occur in smaller densities (population size), have restricted ability to spread, and are limited to one generation per year (in most situations), meaning that resistance can be durable. Cultivars containing the *H1* resistance gene continue to provide almost complete control of *G*. *rostochiensis* populations present in Europe, although populations of *G*. *pallida* that are virulent against the *Gpa5* resistance source have been reported (Mwangi et al., 2019). While host resistance can provide excellent control of PCN, the pyramiding of additional sources of resistance will be required for durable control in the future. The absence of clonal lines of PCN that are virulent or avirulent against specific resistance sources makes phenotyping for breeding of cultivars that contain multiple resistance sources extremely challenging. Fortunately, reductions in the costs of DNA sequencing have facilitated the development of powerful new tools for identification and high‐resolution mapping of *R* genes. For example, combining two enrichment sequencing techniques, one specifically targeted at *R* genes (resistance gene enrichment sequencing. RenSeq; Jupe et al., 2013) and another based on 1,800 single copy genes distributed across the genome (GenSeq), has enabled the mapping of the *H2* resistance gene, which controls the Pa1 pathotype of *G*. *pallida*, to an interval of less than 5 Mb on the potato genome (Strachan et al., 2019). Critically, these techniques also allow the development of KASP markers that can be used for further refinement of the mapping of the gene but that are also compatible with marker‐assisted breeding programmes (Chen et al., 2018).

## 9 CONCLUSIONS

Genomics and transcriptomics have provided an opportunity for a major change in the way that research on PCN is undertaken. We understand more about the biology of PCN and the nature of both the susceptible and resistant host response than ever before. However, there remain numerous key research needs. PCN is entirely dependent on host cues to be able to synchronize its life cycle with host plants. The spectra of chemicals within root exudates that PCN react to is intricate and complicated. Significant progress continues to be made, however, in the identification and understanding of the molecules that PCN responds to in order to hatch and locate host roots. Details of the changes in gene expression that occur in response to these molecules and as part of the restarting of the life cycle have also been characterized. However, we are yet to determine how these two aspects are linked at the molecular level. Details of the mechanisms by which PCN infects plants are emerging but research on host–parasite interactions is still hampered by our inability to genetically transform PCN, especially given the tools that might become available using CRISPR/Cas. We need to understand precisely how syncytia are induced and maintained and why PCN, like other biotrophic plant pathogens, deploys so many effectors during their infection. Understanding the detailed mechanisms behind host resistance to PCN, and whether the same types of novel resistance that do not involve NB‐LRR‐like proteins as described for resistance against soybean cyst nematodes, is also of critical importance towards deploying resistance in the field.

## Data Availability

Data sharing is not applicable to this article as no new data were created or analysed in this study.
